# Seismic Surveys Negatively Affect Humpback Whale Singing Activity off Northern Angola

**DOI:** 10.1371/journal.pone.0086464

**Published:** 2014-03-11

**Authors:** Salvatore Cerchio, Samantha Strindberg, Tim Collins, Chanda Bennett, Howard Rosenbaum

**Affiliations:** 1 Wildlife Conservation Society, Global Conservation Program, Bronx, New York, United States of America; 2 American Museum of Natural History, Sackler Institute for Comparative Genomics, New York, New York, United States of America; 3 New York Aquarium, Brooklyn, New York, United States of America; Heriot-Watt University, United Kingdom

## Abstract

Passive acoustic monitoring was used to document the presence of singing humpback whales off the coast of Northern Angola, and opportunistically test for the effect of seismic survey activity in the vicinity on the number of singing whales. Two Marine Autonomous Recording Units (MARUs) were deployed between March and December 2008 in the offshore environment. Song was first heard in mid June and continued through the remaining duration of the study. Seismic survey activity was heard regularly during two separate periods, consistently throughout July and intermittently in mid-October/November. Numbers of singers were counted during the first ten minutes of every hour for the period from 24 May to 1 December, and Generalized Additive Mixed Models (GAMMs) were used to assess the effect of survey day (seasonality), hour (diel variation), moon phase and received levels of seismic survey pulses (measured from a single pulse during each ten-minute sampled period) on singer number. Application of GAMMs indicated significant seasonal variation, which was the most pronounced effect when assessing the full dataset across the entire season (p<0.001); however seasonality almost entirely dropped out of top-ranked models when applied to a reduced dataset during the July period of seismic survey activity. Diel variation was significant in both the full and reduced datasets (from *p*<0.01 to *p*<0.05) and often included in the top-ranked models. The number of singers significantly decreased with increasing received level of seismic survey pulses (from *p*<0.01 to *p*<0.05); this explanatory variable was included among the top ranked models for one MARU in the full dataset and both MARUs in the reduced dataset. This suggests that the breeding display of humpback whales is disrupted by seismic survey activity, and thus merits further attention and study, and potentially conservation action in the case of sensitive breeding populations.

## Introduction

The coasts and pelagic regions of Africa support a diverse assemblage of marine life, including populations of large whales in various states of recovery from commercial whaling. However, cetaceans in these regions are among the most poorly understood and documented on the globe, with many open questions regarding species presence, distribution, timing of migrations and importance of habitat for critical life functions. At the same time, offshore industrial development (e.g., oil and gas exploration and extraction) along the African coast is increasing rapidly, and occurring in the absence of reliable baseline information about regional cetacean populations, their behaviour, and their response to anthropogenic acoustic exposure [1]. The region is a focal area for new offshore development activities that will generate underwater noise, and also includes important breeding, feeding and migratory habitats for several cetacean species [2], [3]. It is a necessarily important research frontier in the effort to understand, plan for, and mitigate anthropogenic acoustic impact on cetaceans.

Humpback whales (*Megaptera novaeangliae*) in the southern hemisphere are distributed in circumpolar high latitudes during the austral summer and migrate to low latitude breeding areas in the austral winter. The population that winters in the Southeast Atlantic Ocean has a winter distribution along the western coast of Africa from Namibia to Nigeria and the Gulf of Guinea [4]–[7]. Humpback whales are well known for their song, an elaborate male breeding display [8]. Studies have shown that songs are organized into a stereotyped, hierarchical pattern of units, phrases and themes, all males within a population share a common set of themes, and songs gradually change over months and years [8]–[12]. Units are spectrally highly diverse, ranging from <0.5 sec to several seconds in duration, with fundamental frequencies in a broad bandwidth from approximately 30 Hz to over 10 kHz (although predominantly below 1,000 Hz), harmonic energy beyond 24 kHz, and exhibiting complex harmonic structure, rapid frequency modulation and varying amplitude modulation [8], [11], [13] (S.Cerchio, pers. observ.). Maximum source levels of units have been measured ranging from 151 to 173 dB re: 1 µPa (assumed @ 1 m, however not stated in the reference) root mean square (rms) [13]. Phrases are the fundamental pattern of repetition, most analogous to a bird “song” as a comparison [12], and can be simple or complex, containing from 2 to over 20 units and ranging from under 10 sec to over 30 sec in duration [8], [11] (S.Cerchio, pers. observ). Singing occurs predominantly in breeding regions and is believed to be important for male reproductive success [14]–[17]; males also sing to a lesser extent in feeding regions [18]–[20] and during migration [21]. Thus passive acoustic monitoring for the presence of song is a useful indicator of breeding activity, distribution and/or migratory timing. Furthermore, since song is an important breeding display, the impact of anthropogenic activities, either by disturbing singing males or acoustically masking song display, can have potentially negative effects on the reproductive success of individuals and populations.

The impact of anthropogenic noise, particularly in the low-frequency range, on marine mammals has been widely discussed [22]–[27]. Seismic imaging of the seafloor substrata, such as in the exploration for oil and gas reserves, involves the production of intense impulsive sounds to image the upper layers of oceanic crust. Source levels of seismic survey sources have been reported up to 260 dB re: 1 µPa rms @ 1 m [26], or 250 dB re: 1 µPa peak-to-peak [28] with peak spectral levels in the 5 to 300 Hz bandwidth [26] for airgun arrays, and 193 dB re: 1 µPa peak-to-peak in the 30 to 450 Hz bandwidth for sparker sources [28]. Although numerous studies have investigated and documented behavioural changes (e.g., avoidance) of Mysticetes in response to seismic survey pulses [29]–[37], few published studies have specifically assessed the impact of seismic survey operations on vocalization behaviour [28], [38], [39]. To date none have assessed disturbance of displaying male humpback whales in a breeding region.

As part of an assessment of cetaceans offshore of Angola, we collected nine months of continuous passive acoustic data from two offshore locations. This study reports on the presence of humpbacks whales between the months of June and December 2008, as indicated by recorded singing males, and provides evidence that noise introduced by seismic surveys negatively impacts singing activity in this region.

## Methods

### Field Site and Data Collection

Work was conducted in northern Angola off the Congo River mouth outflow (Figure S1). Passive acoustic monitoring was one component of a larger project to assess marine mammal presence around the construction site of the Angola Liquefied Natural Gas (ALNG) plant. Marine Autonomous Recording Units (MARUs) used in this study were developed by the Cornell Bioacoustic Research Program [16], [28],[38],[40] (www.birds.cornell.edu/brp). Data were recorded between March 2 and December 1, 2008 from two MARU locations (labelled 1 and 2 in Figure S1) deployed at approximately 100 meters depth, 24 km and 15 km offshore of the Sereia Peninsula, on the edge of the Congo River Submarine Canyon, and separated by 9.65 km (at coordinates 6.080°S, 12.057°E and 6.046°S, 12.137°E). MARUs were configured to record continuously for approximately 80–100 days with an effective bandwidth of 1,000 Hz (2,000 Hz sampling rate), targeting the sounds of baleen whales, and were deployed three times for a continuous period of nine months. The MARUs had an effective sensitivity of approximately −151.7±3 dB re: 1 V/µPa from 10–1,000 Hz, with an approximately flat (±1 dB) frequency response from 55–585 Hz and nominal dynamic range of 63.2 dB. Recovered data amounted to 11,016 hours of continuous recordings.

### Acoustic and Song Analysis

Recordings were analyzed using software Raven Pro 1.3 or 1.4 (www.birds.cornell.edu/brp/raven). Spectrograms (1024pt Fast Fourier Transform (FFT) with equivalent frame size, Hann window, 75% overlap) were visually scanned for the entire nine month duration, and it was determined that humpback whale song was not present during March to May; song was first recorded in early June and continued through December. A protocol was then developed to assess the number of singing whales recorded throughout the migration and breeding season, as follows. The song typical of the population and year was characterised in terms of phrases and themes using several high signal/noise recordings for whales that sung near a MARU uninterrupted for >1 hour, as well as a single broadband recording (24 kHz bandwidth) of song made from the boat during one deployment. Printed spectrograms of these songs were used to help identify phrases and count singers during the analysis. Although the frequency response of the MARUs did not cover the entire bandwidth of typical humpback whale song, all phrases in the song had some units that were below 1,000 Hz; therefore it was possible to detect and identify all phrase types in the 1,000 Hz bandwidth of the MARU when present. Due to the enormity of the dataset, sub-sampling of the entire second and third deployment periods was completed in order to assess the minimum number of singers present every hour, for every other day from 24 May to 1 December 2008. Spectrograms (512pt FFT, equivalent frame size, Hann window, 75% overlap; spectrogram resolution of 64 ms and 3.9 Hz) and waveform envelopes of the first 10-minute period for each hour were viewed on Raven, with each MARU represented as a different channel in temporally aligned, horizontal panels; the entire 10-minute period was scanned to determine a 1-minute interval in which the most overlapping singers were audible. The number of singers was then visually counted from the spectrograms by assessing the number of directly overlapping units, as well as overlapping phrases (since whales singing different phrases were readily identified) and amplitude differences between singers at varying distances from the MARUs. The counts of singers in the 10-minute periods were used as instantaneous scan samples of the number of singers within detection range at the top of each hour, and considered a metric for the minimum number of singers for that hour, hereafter descriptively referred to as singers/hr for ease of use. When a singer was audible on both MARUs, it was identified as the same individual by recognizable phrases on both MARUs, offset in time as a result of time-of-arrival differences of the signal at each MARU. If a 10-minute period was encountered that contained an obvious close passing boat that raised the foreground noise floor and potentially masked singers, the next 10-minute frame in the hour was reviewed until a 10-minute frame without foreground boat noise was found for measurement. Singer counts were completed by two analysts, with a senior analyst (CB) monitoring the work of the other; since it was relatively straightforward to diagnose the presence of one to three singers, but more challenging to obtain an accurate count when there were >3 singers, all counts of four or more singers were reviewed by the principle investigator (SC).

During the review of recordings for singers, several periods were encountered where offshore seismic survey pulses were detected on a daily basis. Seismic survey pulses were also logged for each MARU when encountered in order to measure Received Level (RL) at the MARU, and care was taken to select (by drawing a Raven selection box around it) a single pulse in the series that did not overlap with other signals in the frequency spectrum over which the pulse was visible on the spectrogram. For many of the lower amplitude pulses, there were higher amplitude sounds (such as whale song units) in the same time slice but different frequency range, and thus peak-to-peak amplitude or broadband RMS (Root Mean Square) measurements would not provide an accurate measure of the RL for the lower amplitude pulse. Therefore we sought an indicator variable that would accurately reflect the variation in pulse RL over the very broad range of amplitude values recorded, and thus appropriately represent the RL for statistical inference. The Peak Power measurement in Raven 1.4 [41] was chosen as the indicator variable, measured with a 1,024 point FFT, equivalent frame size, and Hann window (analysis resolution of 512 ms and 1.95 Hz). The Peak Power measurement provided a spectrogram-based measure of power for the single time/frequency bin (determined by above FFT parameters) containing the highest power spectral density (in dB re: 1 dimensionless sample unit) [41]; this unreferenced measurement was converted into a RL using a calibration constant for the MARU to yield a measurement in dB re: 1 µPa^2^ in a 1 Hz frequency band. Thus, the measurement was not affected by energy present in other frequency bands, and the measured bandwidth was standardized across the range of all pulse bandwidths and amplitudes recorded. The frequency at which the Peak Power occurred, the Peak Frequency, was also recorded, and both measurements were constrained to a minimum low frequency of 15 Hz; thus, if the frequency of Peak Power actually occurred below 15 Hz, only the portion of the pulse above 15 Hz was considered and measured. This was done to account for a conservative bottom range of baleen whale hearing. Once the analysis was completed for every other day across the two deployments, it was determined that all seismic survey pulse activity occurred during two extended periods, from 4 July to 30 July, and from 14 October to 1 December. In order to maximise the dataset for the assessment of impact of seismic survey pulses on singing activity, we collected a complete record of singer counts and presence of seismic survey pulses for all hours of all days during the periods when seismic surveys were active.

### Statistical Analysis

Several variables were statistically assessed to determine their potential influence on singing activity. The dependent variable was the number of singers present each hour, treated separately for each MARU. It was expected that number of singers would vary on a daily basis across the entire period due to timing of the seasonal migration. Seasonality was captured by the Survey Day variable, the number of days since the first singing activity was recorded on 9 June 2008, ranging from 0 to 174. It was also expected that number of singers would vary in a diel cycle as in other breeding regions [16], [40], [42] and was represented by the Hour variable ranging from 0 to 23. Similarly, it has been shown that moon phase significantly influences singing activity [40], hence, Moon Phase was included as a factor variable (New Moon, First Quarter, Full Moon, Last Quarter). The potential interaction between Hour and Moon Phase was also considered (as reported in Sousa-Lima and Clark [40]). To investigate the potential impact of seismic survey pulse activity we considered two variables, namely Peak Power RL (in dB re: 1 µPa^2^ in a 1 Hz bin), and Power Score, a categorical variable 0–5 where each category corresponds to intervals of increasing Peak Power received levels (0 = Seismic survey pulse not detected during the 10 min sample, 1 = 65–75 dB, 2 = 75–85 dB, 3 = 85–95 dB, 4 = 95–105 dB and 5 = >105 dB). Since there was no measurement for Peak Power RL when there was no pulse detected (a Power Score of 0) we used a default value of 60 dB for Peak Power to represent the background noise level in a 1 Hz frequency bin (since the lowest measurement of a pulse was 65.5 dB). We also considered a subset of the data corresponding to the first period of seismic survey activity 5–31 July 2008, to potentially isolate the effect of seismic pulses on singing activity by focusing on a shorter time period during which the variability in singing activity associated with seasonality was reduced compared to that observed during an entire season. The full dataset covering the entire period comprised 3,096 data points (each point representing an assessed 10-minute period), whereas this reduced dataset had 648 data points.

Generalized Additive Mixed Models (GAMMs) were used given their flexibility and capacity for non-linear responses (clearly evident in these data) and options for dealing with temporal correlation (due to the dependence of counts close in time and the likelihood that these represent some of the same singers) [43]. The analysis was completed in R [44], and the auto-correlation function allowed us to visually ascertain the degree of temporal correlation in the data that was treated using an autoregressive model of order one (AR-1) (from the *nlme* library for R [45]). The models were fitted using the *gamm* function from the *mgcv* library [46], which calls the appropriate routine in the *MASS* library [47]. Model selection was based on model diagnostics (residuals vs. linear predictor, histogram of residuals, response vs. fitted values, etc.) [46]. The statistical significance of the terms in the model (based on the approximate p-values produced by *gamm*), Akaike's Information Criterion (AIC) and the adjusted R-squared value were also considered. Cubic regression splines were used to fit the smooth functions (a cyclic smooth was used for the Hour variable to ensure that the first hour matched up with the last hour). Separate smooth functions conditioned on Moon Phase were also considered for the Hour variable to investigate the potential interaction between Hour and Moon Phase. A Poisson distribution and log-link were assumed. The natural variation in singing activity over an entire season (from 0 singers to peak singing activity back to 0 singers) is expected to be much larger than that associated with diel and lunar phase cycles, and potentially also larger than variation that can be attributed to anthropogenic disturbance. Not only are GAMMs able to capture nonlinear and complex relationships, they are also able to detect significant effects for variables with a large range in explanatory power, thus teasing apart natural cycles or human influences that are seasonal, monthly, daily or almost instantaneous.

## Results

**Figure 1:**
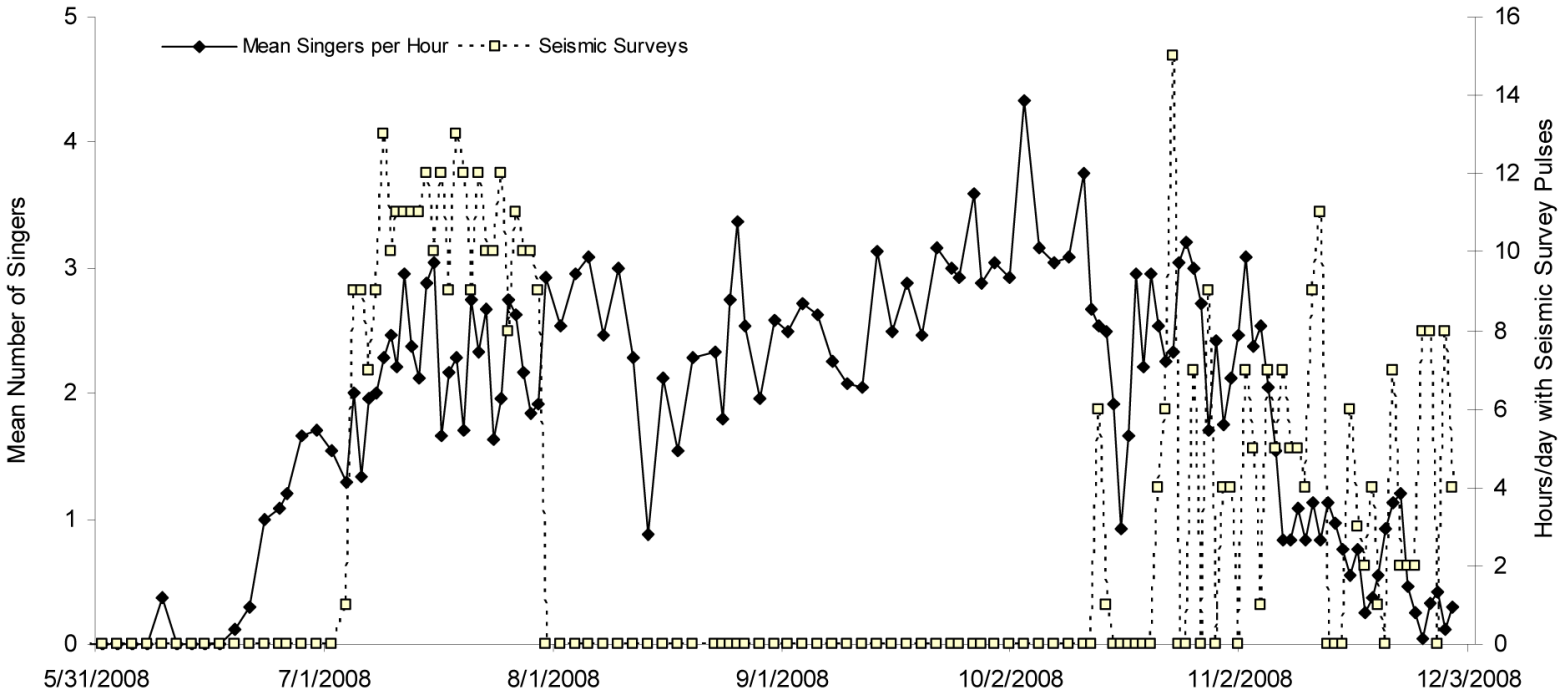
Humpback whale singing activity and occurrence of seismic survey pulses. The number of singers present in the first ten minutes of each hour was counted from spectrographic displays, and thus singing activity is represented by the mean minimum number of singers per hour recorded for both MARU hydrophones combined (accounting for whales heard on both MARUs simultaneously as a single singer). Seismic survey activity is overlaid as the number of hours in a given day during which pulses were detected, scored as present or absent for the same 10-minute periods in which singers were counted.

Humpback whale song was first detected on the MARUs on 9 June 2008, increased steadily throughout June and into July, was constantly present but fluctuated in a non-linear manner until November, and then steadily decreased in occurrence until recording ended in early December (Figure 1). During the primary singing months from 1 July to 31 October, there was a mean of 2.45 (+/− SD 1.07) singers/hr, at least one singer was heard during 98% of all hours examined, 82% of hours had two or more singers and 46% of hours had 3 or more singers. A total of 6,069 individual singer events (accounting for singers heard simultaneously on both MARUs) were logged during 3,106 one-hour periods scored after the onset of singing activity. This value does not represent different individuals because some singers were likely singing during several hours and were thus counted in multiple hours. A detailed analysis of temporal and spatial patterns in singing activity is presented elsewhere [48]. The predominance of song during this period and the high occurrence of chorusing (multiple singers) indicate that this is breeding habitat, as opposed to solely a migratory corridor for which we would expect a pulse of singing activity at the start and end of the season with a quiescent lull in the middle.

### Seismic Survey Pulse Detections

**Figure 2:**
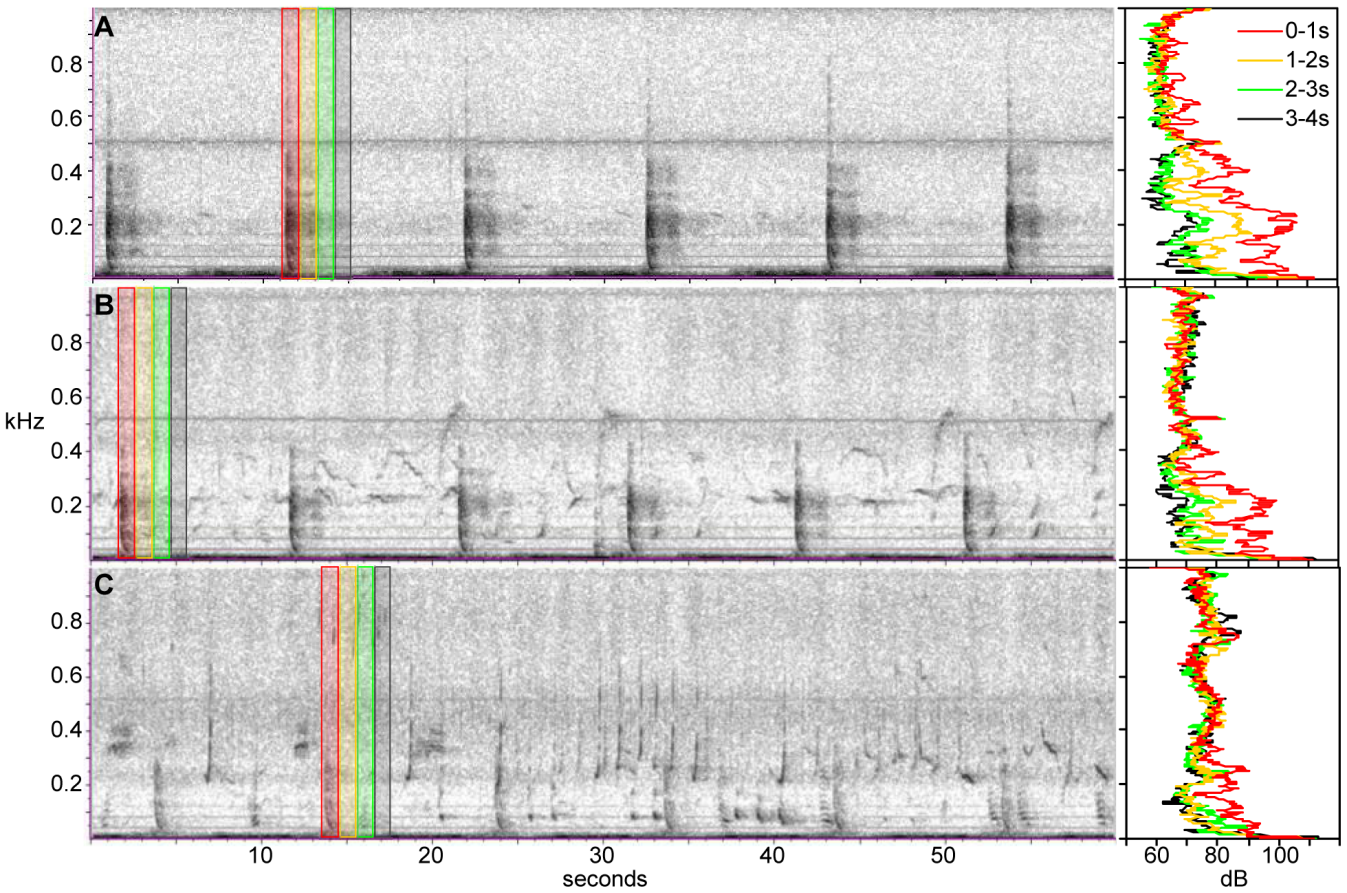
Examples of humpback whale song and seismic survey pulses of varying received level (RL). Each panel represents a spectrogram (FFT 512, same frame size, 75% overlap, Hann window) of a different period recorded on MARU 1. From top to bottom, the panels illustrate a 60-second sequence from a 10-minute sample period in which the measured pulse RL (re: 1 µPa^2^ in a 1 Hz bin, Peak Power) was (**A**) 109.8 dB, (**B**) 101.8 dB, and (**C**) 99.2 dB, representing the upper 1%, 5%, and 10% of all measured pulses, respectively. Power spectral density levels (in dB re: 1 µPa^2^/Hz) are shown to the right of each spectrogram, averaged across four consecutive 1-sec intervals beginning with the onset of the pulse (indicated on the spectrogram with colour coding, i.e., given the onset of pulse = 0 sec, averaged spectra are shown for 0–1 sec, 1–2 sec, etc.). It can be seen that pulses were approximately 10–11 seconds apart with most spectral energy in the 0–500 Hz bandwidth; reverberation lasting approximately 2 seconds was evident only on the loudest 1% of pulses, whereas 90% of pulses had a lower RL than illustrated in the bottom panel, with no detectable reverberation beyond 1 second.

Seismic survey pulses were detected in a total of 449 hour periods during 50 days. These were divided into two distinct periods, with seismic surveys detected on all 27 days between 4 July and 30 July, on 33 days between 14 October and 1 December, and never outside of these periods (Figure 1). During the July surveys, pulses were detected during 7 to 13 hours each day and were fairly consistent across the 27 day period; during the longer late season period the occurrence was more variable and sporadic, with pulses heard during 0 to 15 hours each day across the 49 day period. Seismic pulses were more frequently detected on MARU 1, during 444 hours compared to 243 hours on MARU 2, and were regularly recorded coincident with humpback whale song (as depicted in Figure 2). An important concern to address is the possibility that masking of humpback whale song by the seismic pulses may have hindered the ability of analysts to detect and accurately count singers. In order to assess the potential for masking, we have provided examples illustrating a sequence from three separate 10-minute periods in which the measured Peak Power RL fell within the upper 1%, 5% and 10% of all measured RLs (Figure 2a, b and c, respectively). A small degree of smearing (reverberation) is evident in the upper 1% example, extending approximately 2 sec into the 11 sec interval between pulses, but nearly gone after 2 sec (see spectra in Figure 2a, showing a peak of approximately 110 dB during the 0–1 sec interval after pulse onset, dropping approximately 10 dB by the 1–2 sec interval, and dropping an additional 5–10 dB by the 2–3 sec interval to near background level). This effect is still present in the upper 5% example but greatly reduced (see spectra in Figure 2b for 0–1 sec and 2–3 sec intervals), and nearly absent in the upper 10% example where reverberation is evident for less than 1 sec after the arrival of the pulse signal (see spectra in Figure 2c for 0–1 sec interval). Therefore, masking is not likely to have affected detection of singers, since even in the loudest 1% of measured pulses, most of the temporal domain is free of masking (Figure 2a, in which a single faint singer is detectable despite the relatively high amplitude of the pulse), and for 90% of measured pulses there was little if any detectable reverberation (Figure 2c). Furthermore, the complexity of pattern and temporal consistency in utterance of humpback song further reduces the potential of missing singers, since the human reviewers were not simply looking for single units of song, but rather detecting the patterns of multiple units that compose phrases; humpback song units were often greater than 1–2 sec (the length of reverberation) and phrases typically greater than 10 sec (the period of seismic pulse repetition), so we do not consider masking a potential bias for accurately counting the number of singers.

**Figure 3:**
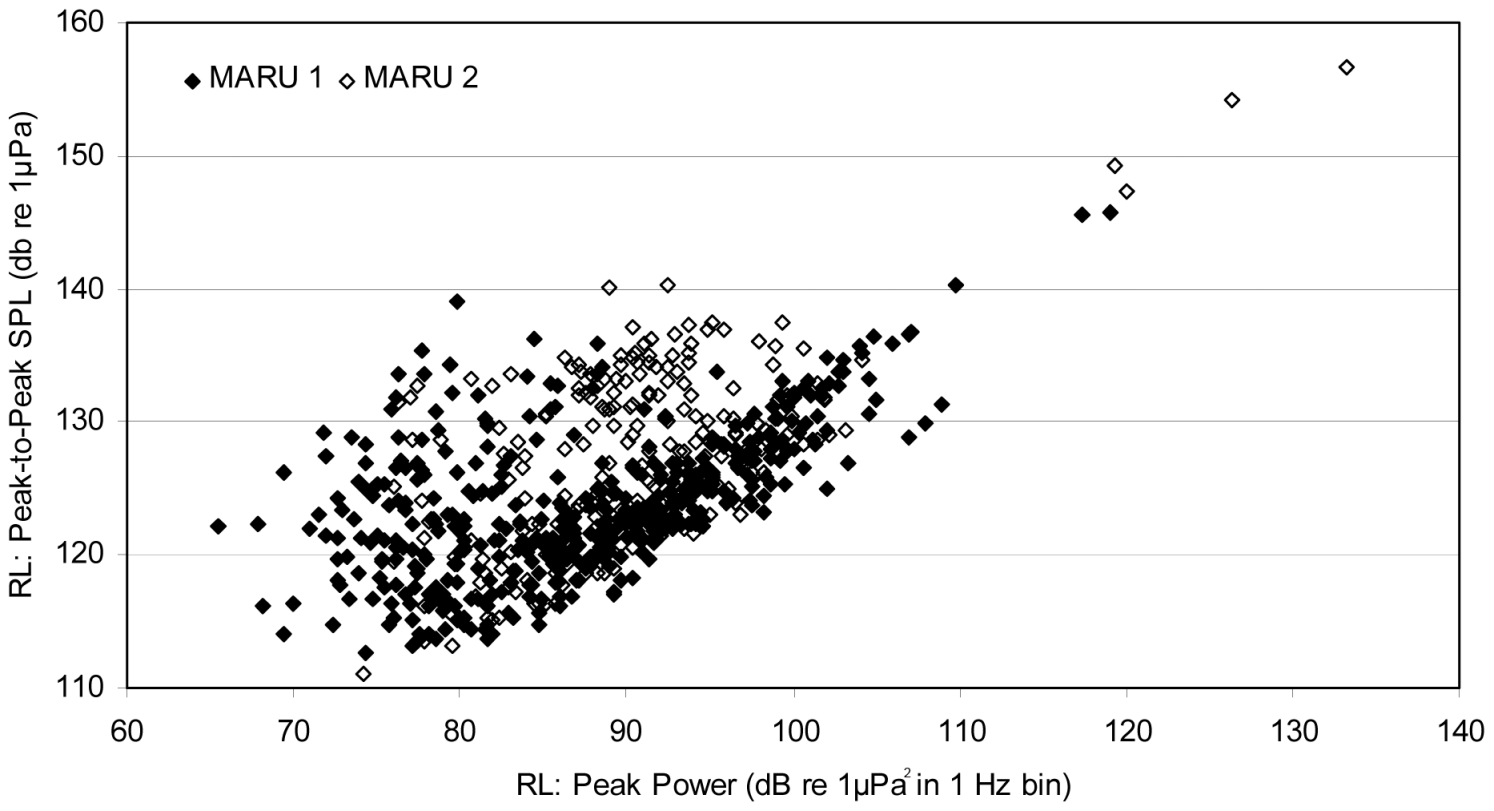
Received level (RL) of seismic survey pulses. Plotted is the relationship of the measured Peak Power variable (in dB re: 1 µPa^2^ in a 1 Hz band for the frequency bin with the highest power; used in the GAMM analysis) to the Peak-to-Peak amplitude (in dB re: 1 µPa) for all measured seismic survey pulses.

The measured Peak Power in a 1 Hz bin ranged from 65.5 to 133.2 dB re: 1 µPa^2^/Hz, with a “logarithmic mean” (averaged after dB values were converted to the linear pressure domain) of 106.5 dB; this mean value falls within the top 1.9% of all values in the sample, and thus it substantially biases the reported “average” towards more intense sounds. Therefore the sample arithmetic mean of 88.4 (+/− SD 8.7) dB and median value of 88.7 dB may be considered more useful sample statistics as a measure of central tendency. Since the Peak Power metric was chosen as an indicator variable, and has little relationship to more familiar measures, the Peak-to-Peak sound pressure level (SPL) was also measured in order to assess the broadband RL of seismic survey pulses. Peak-to-Peak SPL has been recommended by multiple sources as an appropriate metric for measuring and reporting RL of impulsive signals, specifically seismic survey pulses, and often preferable to RMS SPL [24], [26], [49], [50]. Peak-to-Peak SPL ranged from 111.1 to 156.7 dB re: 1 µPa, with a logarithmic mean of 133.2 dB, a sample arithmetic mean of 125.1 (+/− SD 6.2) dB, and a median of 124.2 dB. It was noted that the single most intense pulse recorded, with a Peak Power of 133.2 dB re: 1 µPa^2^/Hz and a Peak-to-Peak SPL of 156.7 dB re: 1 µPa, displayed obvious clipping (truncation) of the waveform, indicating that this signal surpassed the maximum measureable amplitude and overloaded the system. The next most intense pulse (126.3 dB re: 1 µPa^2^/Hz Peak Power, and 154.1 dB re: 1 µPa Peak-to-Peak), and all remaining pulses showed no indication of waveform clipping. The Peak Power in a 1 Hz bin was plotted against the Peak-to-Peak SPL for each measured pulse (Figure 3), and there was a strong linear relationship with Peak Power for most pulses; however, there was a scatter of pulses between 70–105 dB Peak Power with a Peak-to-Peak SPL greater than predicted by the linear relationship (Figure 3). These represent seismic pulses during which there was a different and confounding sound source of greater amplitude in the same time bin but different frequency band (e.g., a humpback whale signal), thus demonstrating the need to use Peak Power in a 1 Hz frequency bin as the indicator variable for RL of pulses. Peak Frequency ranged from 15.6 to 406 Hz, with a mean of 123.8 (+/− SD 68.2) Hz, well within the predominant communication band of humpback whale song. Pulses received at MARU 2 tended to have Peak Power at the higher end of the frequency range, greater than 100 Hz (mean of 165.5 +/− SD 60.2), whereas pulses with Peak Power between 15 and 100 Hz were detected predominantly on MARU 1 (mean of 101.0 +/− SD 61.2 Hz) (Figure S2). The loudest pulses were heard on MARU 2 during a single day, from 11:00 to 14:00 on 14 October, when a survey vessel was apparently operating particularly close to the unit and produced a Peak-to-Peak RL in excess of 156.7 dB. Despite this subset of large value measurements (see Figure 3), the (sample arithmetic) mean Peak Power measured at the MARUs was relatively similar, 87.3 (+/− SD 9.2) dB re: 1 µPa^2^/Hz for MARU 1, and 90.4 (+/− SD 9.2) dB re: 1 µPa^2^/Hz.

### GAMM Results

The models considered included all combinations of the Survey Day, Hour, Peak Power and Moon Phase variables. Namely, the full model with all four variables, models with one of these variables removed, models with two variables and finally single variable models. Table 1 shows the top five models ranked by AIC value for each MARU for both the full and reduced dataset, where the latter corresponds to the first period of seismic survey activity. We omit the results for Power Score and only discuss those for Peak Power RL, as models including either of these variables gave almost identical results in terms of AIC value, significance of the variable, and adjusted R-squared value. All the model covariates turned out to be significant (at the 5% level or greater) for several top-ranked models. Conditioning the Hour smooth function on the Moon Phase factor did not lead to any significant improvements in the models.

**Table 1:** Results for Generalized Additive Mixed Models fit to data from MARU 1 and MARU 2.

Dataset	MARU	Model No.	Survey Day	Hour	Peak Power	Moon Phase	AIC	Delta AIC	R-sq (adj)
Full	1	1	***	**		***	4,586.99	0.00	0.482
		2	***	**	*	***	4,588.59	1.61	0.484
		3	***			***	4,593.16	6.18	0.471
		4	***		*	***	4,594.79	7.80	0.473
		5	***	*** #	**	***	4,596.15	9.16	0.485
	2	1	***			***	9,019.85	0.00	0.156
		2	***		**	***	9,034.08	14.24	0.159
		3	***	*** #	**	***	9,093.03	73.18	0.184
		4	***	***		***	9,097.59	77.75	0.187
		5	***	***	**	***	9,112.22	92.38	0.190
Reduced	1	1		**	*	***	571.74	0.00	0.090
		2	**	**			572.65	0.91	0.091
		3		**			572.86	1.12	0.041
		4	**	**	*		573.00	1.26	0.098
		5		**	*		573.34	1.59	0.046
		6^1^			*	***	573.60	1.85	0.050
	2	1			**		1,476.30	0.00	0.014
		2			**	ns	1,484.39	8.08	0.015
		3	ns				1,485.61	9.31	0.030
		4	ns			ns	1,494.38	18.07	0.037
		5	ns		**	ns	1,494.42	18.12	0.052

“Dataset”: results are shown for a GAMM analysis using data from the entire period of singing activity, 9 June to 1 December 2008 (“Full”), and the reduced dataset during only the first period of seismic activity, 5–31 July 2008 (“Reduced”) for each of the two hydrophones (“MARU” 1 and 2). For each of the potential covariates (“Survey Day”, “Hour”, “Peak Power” and “Moon Phase”, see text for detailed descriptions) a blank cell indicates the variable was not included in the model, otherwise the significance level for the variable is shown (‘***’<0.001, ‘**’<0.01, ‘*’<0.05, and ‘ns’ indicates non-significant terms at the 5% level in the models). The hashes (‘#’) indicate models where Hour was conditioned on the Moon Phase factor variable. “AIC”: Akaike's Information Criterion value for each model; “Delta AIC”: the difference in AIC between the model under consideration and the model with the minimum AIC; and “R-sq (adj)”: the adjusted R-squared value, which is the proportion of variance explained. All models include the AR-1 correlation structure.

1 Six rather than the top five models are shown for MARU 1, as these all have a Delta AIC value of less than 2.

The value of auto-correlation function at different time lags indicated significant auto-correlation in the data. For example, for MARU 1 and 2 for the top model using the full dataset, the correlation between residuals separated by one time unit (adjacent samples separated by one hour) is 0.513 and 0.475, respectively; by two it is 0.513^2^ = 0.263 and 0.475^2^ = 0.226, respectively. The value of the correlation between residuals is fairly similar across all models considered. All top models ranked by AIC include the AR-1 correlation structure to deal with the temporal correlation, as this produced a significant reduction in AIC value and improved diagnostic plots.

The top-ranked model, and models with a difference in AIC value of <2 from the top-ranked model, are all plausible candidates [51]. Thus, using the full dataset, for MARU 1 the model with Survey Day, Hour and Moon Phase (AIC = 0.00) has approximately equal weight in the data to the model that also includes Peak Power (AIC = 1.61; Table 1). Although seasonality (Survey Day) in the full dataset explains the largest proportion of the variation in the number of humpback singers and to a lesser extent time of day (Hour) and moon phase, the Peak Power variable is significant and indicates that singer numbers are reduced with an increase in received levels of seismic survey pulses. For MARU 2 the top ranked model included only Survey Day and Moon Phase, with a much larger difference in AIC values between the top-ranked and the remaining models. Although the Peak Power (and the Hour) variable were significant, they were not in the top-ranked model, likely due to the overwhelming influence of Survey Day in the full dataset (see results for reduced dataset below). For Peak Power this may also be explained in part by the fact that most of the seismic survey activity was detected offshore and a smaller proportion of seismic survey pulses were received by MARU 2 located further inshore.

**Figure 4:**
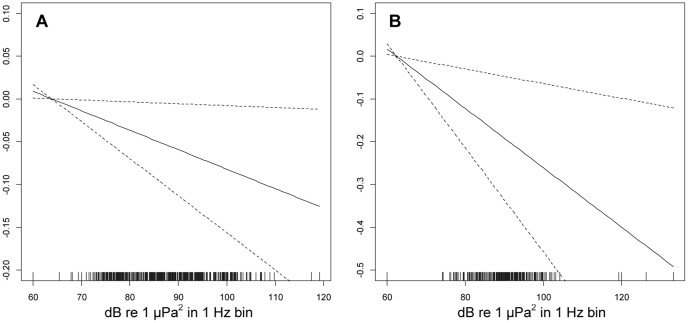
Impact of seismic survey pulse RL on humpback whale singing activity for the full dataset. Generalized Additive Mixed Models of the number of humpback whale singers with smooth terms for the dependence on Survey Day, Hour, Moon Phase and Peak Power fitted for each of the MARUs; plots show the estimated conditional dependence of humpback whale singer numbers on Peak Power for (**A**) MARU 1 and (**B**) MARU 2. The x-axis in each plot shows Peak Power, describing received level of seismic survey pulse (in dB re: 1 µPa^2^ in a 1 Hz frequency bin) with a rug plot (short vertical bars) indicating the Peak Power values of observations. The y-axis, with scale selected optimally for each plot, shows the contribution of the smooth of Peak Power to the fitted values of singer number. Estimates (solid lines) are shown with 95% confidence bands (dashed lines), indicating a significant downward trend in singer number with increasing pulse RL.

There was a significant trend for fewer singers during periods with higher received levels of seismic survey pulses at both MARU 1 (*p*<0.05, effective degrees of freedom (edf) = 1) and MARU 2 (*p*<0.01, edf = 1) (Table 1, Figure 4). The estimated reduction in number of singers was greater for MARU 2, possibly due to several extreme values corresponding to the highest measurements for received levels documented. Figure 4 shows the estimated smoothers for Peak Power for the GAMM models for number of humpback whale singers with covariables Survey Day, Hour, Moon Phase and Peak Power for MARU 1 and MARU 2 for the full dataset (although for MARU 2, this model differed considerably in terms of AIC value from the top-ranked model, its results are shown to permit a contrast between the MARUs). The Survey Day variable was highly significant (*p*<0.001, edf values of 8.20 and 7.59 for MARU 1 and MARU 2, respectively) for both MARUs, with multi-modal seasonal distributions; there was also significant diel variation (*p*<0.01, edf values of 3.15 and 3.64 for MARU 1 and MARU 2, respectively) for both MARUs, with peak singing activity during the night (see Figure S3, for estimated smoothers for Survey Day and Hour). Using “New Moon” as a reference level, the model results indicated that there was a significant reduction (*p*<0.001) in singers during brighter moon phases for both MARUs.

The reduced dataset (5–31 July 2008) encompassed a period for which singing had become relatively steady after the early season “ramp-up”, and seismic survey pulses were recorded every day (Figure 1), thus allowing an assessment of variable effects without the overwhelming influence of seasonal changes evident in the full dataset. In the reduced dataset Survey Day and Moon Phase were frequently not significant (Table 1). The former probably because the period corresponded to a period of relative seasonal stability in singer numbers and the latter possibly due to the lack of replication for this variable over the shorter time period (one month). For the reduced dataset, for MARU 1 the top-ranked model includes Hour, Peak Power and Moon Phase, and Peak Power is included in four of the six top-ranking models with difference in AIC value <2. For MARU 2 the top-ranked model includes only Peak Power, and furthermore is the only plausible model with the next model (which also includes Peak Power) having a difference in AIC value of 8.08 from that top-ranked model. Similar to the full dataset, the smoothers for Peak Power for these reduced dataset models (see Figure S4) again show a significant trend for fewer singers during periods with higher received levels of seismic survey pulses at both MARUs (*p*<0.05 for MARU 1, *p*<0.01 for MARU 2, with edf = 1 in both cases, Table 1). The estimated reduction in number of singers was again greater for MARU 2, possibly due to the extreme values of Peak Power recorded. Overall, the results of the reduced dataset, which attempted to partially isolate the effect of seismic pulse RL from seasonality, reinforced the conclusion that there was a real and detectable negative effect of increasing seismic pulse RL on singing activity.

In contrast to the models over the full dataset where the better models for MARU 1 and 2 had an adjusted R-squared value of between 0.471–0.484 and 0.156–0.190, respectively, this value plummets in the reduced data models to between 0.041–0.091 and 0.014–0.144, respectively (Table 1). In particular, the relative percentage of the variability in the number of humpback whale singers explained by Survey Day using the full dataset was just over 45% and 18% for MARU 1 and 2, respectively; this percentage was always less than 1% for Hour, Peak Power, and Moon Phase, except for MARU 2 where the percentage of the variability explained by Peak Power approached 2%. For the reduced data, not surprisingly, these percentages for Survey Day plummeted to less than 1% and approximately 5% for MARU 1 and 2, respectively; with the other variables gaining a little in relative explanatory power with percentages of just under 4% and just over 7% for Hour, similarly just under 1% and just over 1% for Peak Power, and just over 4% and 1% for Moon Phase, for MARU 1 and MARU 2, respectively. This highlights the explanatory power of the seasonality (Survey Day variable) in the full dataset, and also demonstrates that the effect of the seismic survey pulse RL was on par with the remaining ecological variables (Hour and Moon Phase). Although the proportion of variance explained is considerably better for the models on the full dataset, especially for MARU 1, there is indication that certain key variables are missing, for instance variables associated with social context, density of individuals, and potential disturbance due to other anthropogenic influences, such as boat traffic and noise (as reported by Sousa-Lima and Clark [40]).

## Discussion

The presence of oil and gas exploration activities in this region and globally, and the potential acoustic impact that anthropogenic noise sources may have on sensitive species are increasing areas of study and concern for industry, governments, biologists and conservationists [23], [25]. The intense pulses produced by seismic surveys clearly have the potential to cause direct or behaviourally mediated physiological harm at close distances [34], but more subtly at longer distances there exists the potential of disturbing animals and altering important behaviours, as well as masking acoustic signals and negatively affecting communication. We have demonstrated with our GAMM analysis that the seismic survey pulses recorded during our study period had a negative effect on the number of detectable singers in this region, with singing activity declining with the presence and increasing received levels of seismic survey pulses at the MARUs. It appears that whales are ceasing to sing, or moving to other areas to sing when seismic surveys are being conducted in relatively close proximity. We emphasize that this is documentation of disturbance of a breeding display for a baleen whale on a breeding ground, and thus has implied potential for affecting mating behaviour and success. The influence of seismic survey pulses was not as strong or pronounced as the influence of seasonality, or in some cases the time of day; however, given the natural variation due to migration over an entire six-month season, from the first to last passage of singing whales, and the diel trends that have been noted in other studies, it is expected that these natural cycles would have a more prominent statistical effect. Additionally, the influence of the seismic survey pulses was in many cases on par with ecological variables, albeit small but nonetheless detectable and significant. The relatively small amount of variation explained does not lessen the implication of a significant reduction of singing activity in response to an anthropogenic sound source. Moreover, this study was an opportunistic “natural experiment” as opposed to a study specifically designed to test the influence of, or disturbance caused by seismic survey pulses; with only two sensors and no prior knowledge of the seismic surveys, we did not have the ability to locate the singers or the seismic survey vessel, estimate the source level of the pulses, the distance between the source and potentially impacted singers, or the received level of the pulses at the singers. Since we could not constrain the analysis to only singers nearest the sound source or condition on distance from the source, the design was less than optimal for the tests that were attempted, the result being a reduced sensitivity to be able to detect effects. Therefore, that a significant effect was in fact detected, is rather remarkable and suggests that it could be more pronounced than indicated by this analysis.

Our results indicating a decrease in humpback whale singing activity in the vicinity of seismic surveys is consistent with recent studies in other Mysticetes assessing an acoustic response to intense anthropogenic noise. Castellote *et al.* [38] recently demonstrated that fin whale singing activity and acoustic features were effected by the presence of seismic survey airgun operations in the western Mediterranean Sea. After the onset of seismic surveys there was a significant decrease in the number of singing fin whales and the received levels of song units, as well as a shift in bearing to singers, suggesting that animals responded by moving out of the area; furthermore the effect was prolonged and noticeable for 14 days after the cessation of the seismic survey. Blackwell *et al.* [39], compared calling rates of bowhead whales in the Alaskan Beaufort Sea in close proximity vs. distant to seismic survey airguns, and found significant decreases in calling rates near the airguns; however, the authors were unable to determine if the observed effect was the result of individual whales ceasing to call or moving out of the area. Although no available studies have assessed the effect of seismic surveys on humpback whale singing activity, Risch *et al.* [52] recently documented a decrease in daily detections of humpback whale song recorded off the coast of Massachusetts (a feeding region) in response to a distant anthropogenic sound source used for imaging fish shoals over a 100 km diameter region. The sound source assessed was composed of three distinct narrow band (approximately 50 Hz) FM pulses centred at 415, 734 and 949 Hz, with pulse duration of approximately 1 sec, and thus were quite different than broadband seismic survey pulses. Risch *et al.* [52] concluded that humpback singers ceased singing during the period of transmission of the pulses over 100 km away, when the RL of the lowest frequency pulse reached an estimated nominal signal excess of 12 dB, a relatively low value, and discussed the implications of such a sensitive response. In another study assessing a narrowband signal, Miller *et al.* [53] observed that five out of 16 singing humpback whales ceased singing in response to controlled playback of low frequency active (LFA) sonar, however, the cessation response was not tested statistically.

Di Iorio and Clark [28] also reported a significant impact of seismic survey activity (a sparker impulse source) on the non-song calling behaviour of blue whales in the St. Lawrence Estuary, however in this case the effect was in the opposite direction noted by our and the above cited studies. Blue whales in the region had a significant tendency to call more frequently during days when seismic surveys were present compared to when they were not present, as well as during hours within days when surveys were present. They interpreted this to be a response on the part of blue whales to compensate for the increased noise levels with greater repetition and redundancy in their signalling, in accordance with expectations from information theory. The difference in response observed in Di Iorio and Clark [28] and our and other studies may be related to differences between social communication used by both sexes (calls recorded in the blue whale study) and song as a male-specific broadcast breeding display (the subject of our study); partly congruent with this, MacDonald *et al.* [54] documented that a blue whale ceased to sing when it came within 10 km of a seismic survey. However, it is noted that Blackwell *et al.* [39] documented a decrease in bowhead whale “calls”, assumed to be social communication and not song due to the time of year (although it is not explicitly defined in the study), in response to seismic survey activity, whereas Miller *et al.* [53] documented lengthening of humpback songs for those individuals that did not cease singing in response to LFA signals, and also suggested a compensation mechanism to increase redundancy similar to that suggested for blue whale calls [28]. To add further complexity to this still relatively small body of literature, Melcon *et al.* [55] recently demonstrated a decrease in blue whale calling in the Southern California Bight (assessing a call associated with foraging, uttered by both sexes) due to presence and increasing RL of mid frequency active (MFA) sonar, congruent with Blackwell et al. [39] but opposite from Di Iorio and Clark [28]. It is noteworthy that the MFA sonar had a bandwidth (approximately 3–5 kHz) entirely non-overlapping with the blue whale call (25–100 Hz) [55]. Therefore, the manner and mechanism of acoustical response by Mysticetes to anthropogenic sound sources may vary dependent on a complex set of variables, including type of source, species, individual, functionality of vocalization, and social context. Clearly this is an important and growing field of study that demands further attention, assessing more subtle behavioural responses (such as vocal activity) in an effort to move beyond more simple assessments of avoidance and spatial distribution.

It is impossible from this study to determine whether the documented decrease in number of humpback whale singers would translate into detrimental impacts on individuals or the population. We can only report that the negative effect on singing activity exists. Songs of humpback whales are breeding displays, and there is good evidence indicating that singing is important in male breeding strategy [16], [17], so it is likely a critical component of male reproductive success [15], [56]. It is therefore possible that disruption of this breeding display or displacement of singing males to less preferred breeding sites could have significant adverse impacts on individual males by negatively impacting their chances to obtain mates. It is conceivable that at some threshold of numbers of impacted individuals, this could translate into adverse impacts at the population level.

With the incidence of seismic exploration in offshore waters increasing around the globe, we find this a reason for concern, particularly for many regions such as Africa where operations are being conducted in known breeding habitat for baleen whales throughout breeding seasons. Currently, in many nations globally there are no regulations governing seismic exploration in relation to marine mammals; recently, the IUCN highlighted the lack of significant interventions and measures to address anthropogenic ocean noise in Africa, recommending among other measures that seismic surveys be restricted to low-risk areas and times of years, and the implementation of international best practices and standards [57]. In Africa and other regions without specific regulation, exploration companies are not obliged to follow guidelines, as have been established in countries such as the U.K. (see the Joint Nature Conservation Committee, JNCC, guidelines; available at http://jncc.defra.gov.uk/page-1534) or the U.S.A. (see Bureau of Ocean Energy Management, BOEM [58], [59]). In our dataset, fully 49.3% of detected pulses occurred during night-time hours when it was not possible for Marine Mammal Observers (MMOs) to search for potentially impacted cetaceans in the vicinity of the survey vessel; this is not recommended by JNCC guidelines, unless there is a Passive Acoustic Monitoring system being operated, something for which we can not assess compliance in our study.

Moreover, this anthropogenic impact is one of several that is known to affect humpback and other Mysticete whales (e.g., fisheries by-catches, ship-strikes, climate-related changes in food resources), with the consequences of a cumulative affect unknown but assumed. Our finding therefore underscores the need to further investigate and test for the effects of such disturbance, the need to improve available data for other species that might also be at risk (particularly in geographic regions such as this), and the need to consider more effective regulations and monitoring of seismic exploration that takes account of breeding seasons in baleen whale breeding areas.

## Supporting Information

Figure S1 **Study site off northern Angola.** Positions of Marine Autonomous Recording Units (MARUs) deployed off Angola at the Congo River outflow (MARUs 1 and 2), deployed ca. 24 km and 15 km offshore, respectively, near the edge of the Congo River Submarine Canyon. These MARUs recorded continuously at a sample rate of 2,000 Hz, during three months from 2 March to 1 December 2008, in three deployments of 81, 88 and 101 days.
(TIF)

Figure S2 **Received levels (RL) and frequencies of seismic survey pulses.** Distributions are shown for (**A**) Peak Power, the RL of the seismic survey pulse (in dB re: 1 µPa^2^ in a 1 Hz frequency bin); and (**B**) Peak Frequency, the frequency at which the Peak Power occurred, for all seismic survey pulses measured for MARU 1 and MARU 2.
(TIF)

Figure S3 **Seasonal and diel affects on humpback whale singing activity for the full dataset.** Generalized Additive Mixed Models of the number of humpback whale singers with smooth terms for the dependence on Survey Day, Hour, Moon Phase and Peak Power fitted for each of the MARUs; the plots show the estimated conditional dependence of humpback whale singer numbers on: Survey Day, the number of days since the first singing activity was recorded (x-axis), for (**A**) MARU 1 and (**B**) MARU 2; and Hour (x-axis), the diel cycle in hours, for (**C**) MARU 1 and (**D**) MARU 2. The y-axis, with scale is selected optimally for each plot, shows the contribution of the smooth term to the fitted values. Estimates (solid lines) and 95% confidence bands (dashed lines), with a rug plot indicating the covariate values of observations (short vertical bars along each x-axis), are shown.
(TIF)

Figure S4 **Impact of seismic survey pulse RL on humpback whale singing activity for the reduced dataset.** Generalized Additive Mixed Models of the number of humpback whale singers with smooth terms for the dependence on Hour, Peak Power and Moon Phase for MARU 1 and only Peak Power for MARU 2, when restricting data to the first period of seismic activity 5–31 July 2008. Shown is the estimated conditional dependence of humpback whale singer numbers on Peak Power for (**A**) MARU 1 and (**B**) MARU 2. The x-axis in each plot shows Peak Power, describing received level of seismic survey pulse (in dB re: 1 µPa^2^ in a 1 Hz frequency bin) with a rug plot (short vertical bars) indicating the Peak Power values of observations. The y-axis, with scale selected optimally for each plot, shows the contribution of the smooth of Peak Power to the fitted values. Estimates (solid lines) are shown with 95% confidence bands (dashed lines).
(TIF)
