# Supplementary figures and images for: Seismic Surveys Negatively Affect Humpback Whale Singing Activity off Northern Angola
Source: PLoS One. 2014 Mar 11;9(3):e86464. doi: 10.1371/journal.pone.0086464 (PMC3949672; doi:10.1371/journal.pone.0086464)

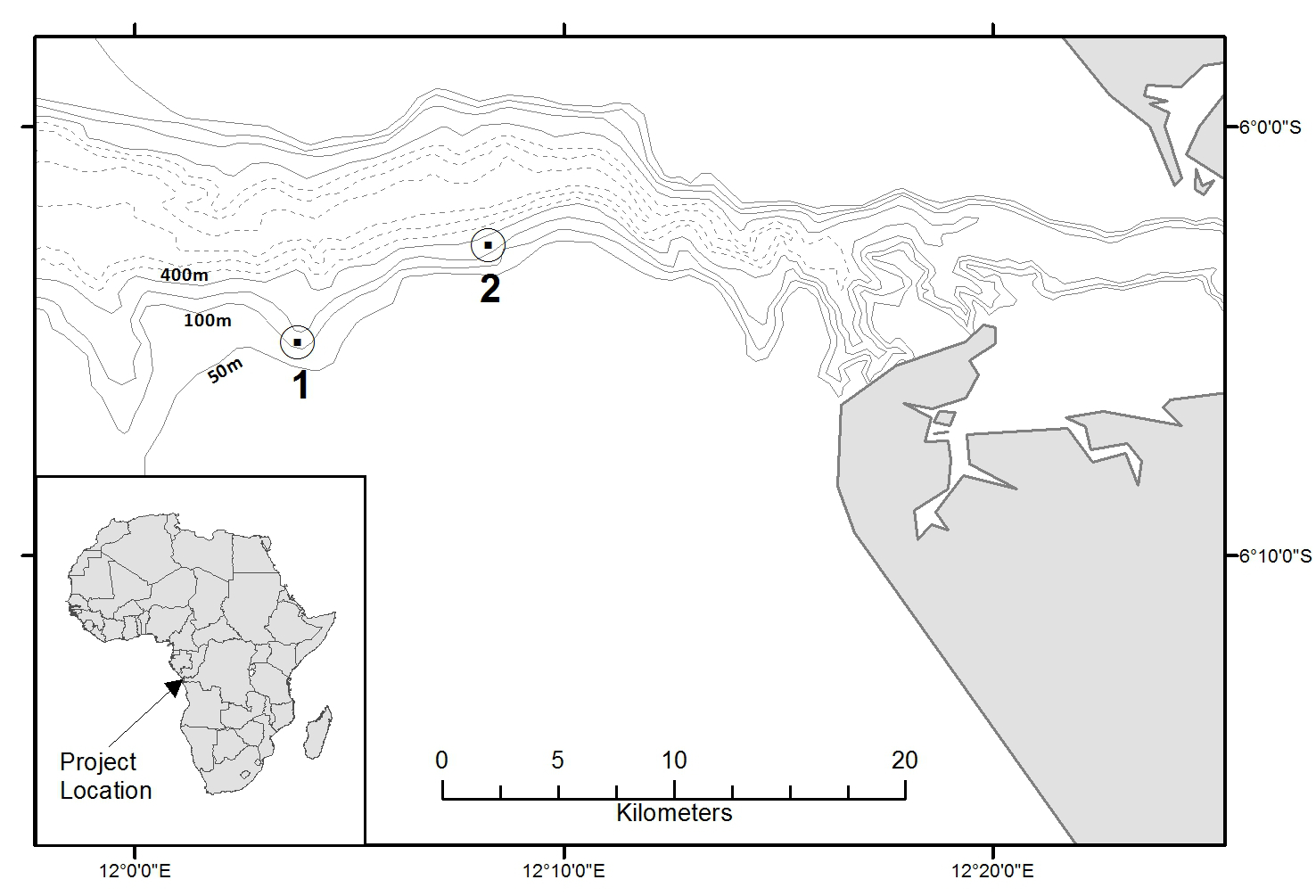

Supplement: Figure S1 — Study site off northern Angola. Positions of Marine Autonomous Recording Units (MARUs) deployed off Angola at the Congo River outflow (MARUs 1 and 2), deployed ca. 24 km and 15 km offshore, respectively, near the edge of the Congo River Submarine Canyon. These MARUs recorded continuously at a sample rate of 2,000 Hz, during three months from 2 March to 1 December 2008, in three deployments of 81, 88 and 101 days. (TIF) [file pone.0086464.s001.tif]

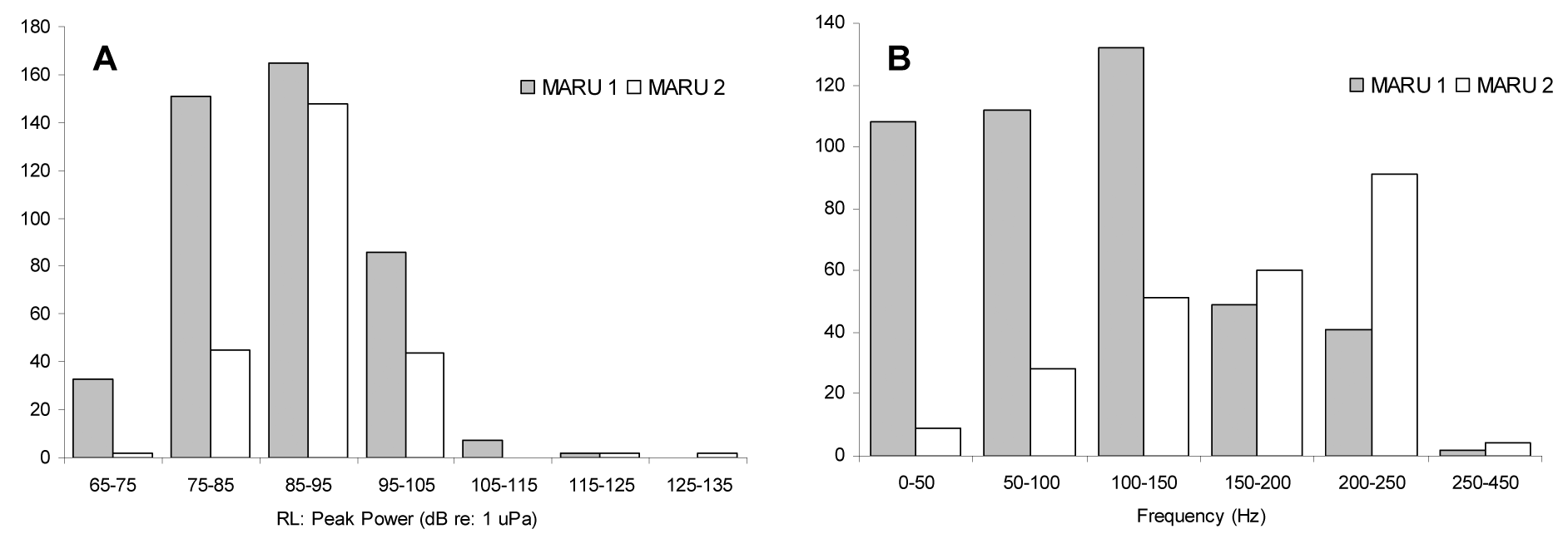

Supplement: Figure S2 — Received levels (RL) and frequencies of seismic survey pulses. Distributions are shown for (A) Peak Power, the RL of the seismic survey pulse (in dB re: 1 µPa2 in a 1 Hz frequency bin); and (B) Peak Frequency, the frequency at which the Peak Power occurred, for all seismic survey pulses measured for MARU 1 and MARU 2. (TIF) [file pone.0086464.s002.tif]

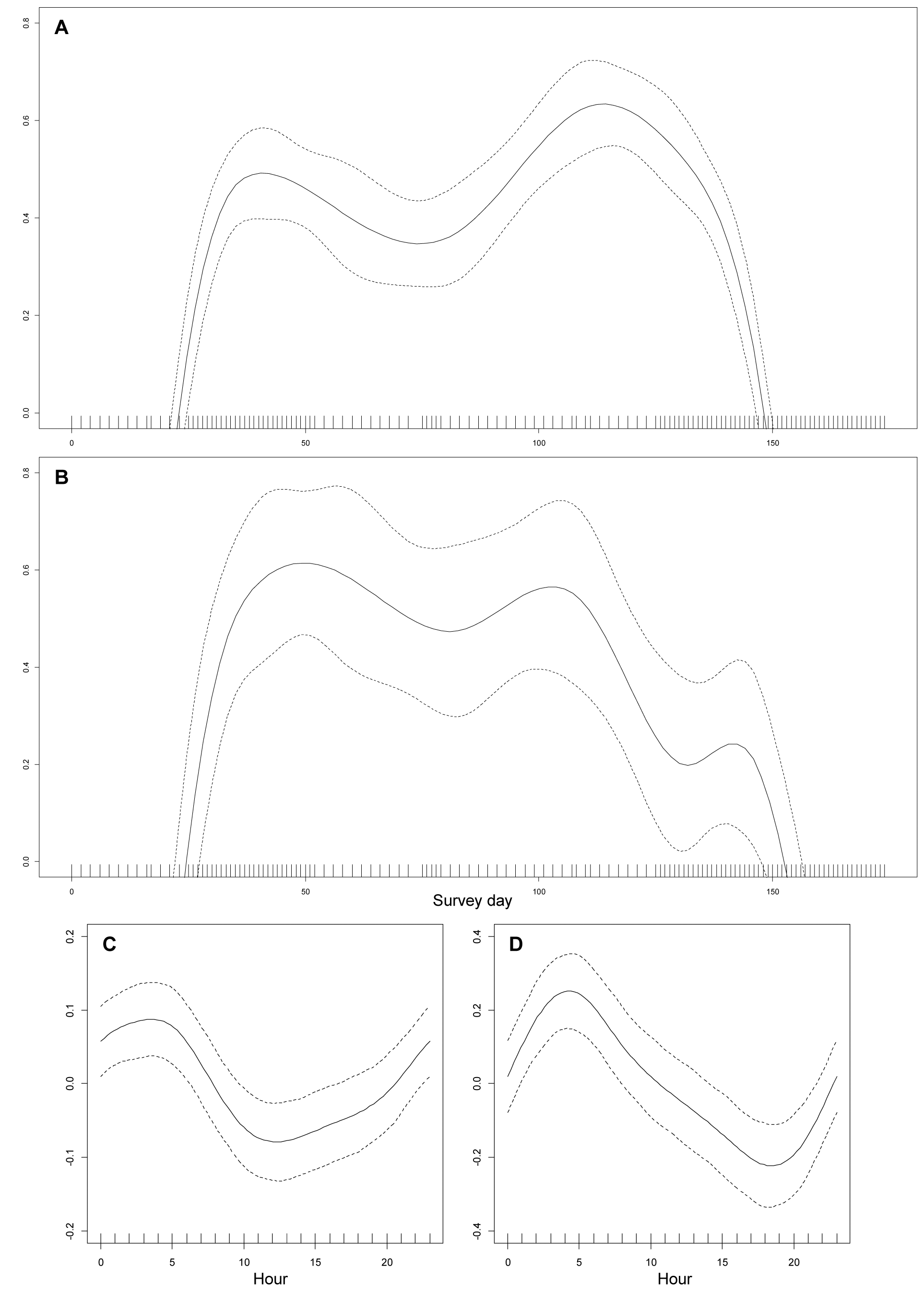

Supplement: Figure S3 — Seasonal and diel affects on humpback whale singing activity for the full dataset. Generalized Additive Mixed Models of the number of humpback whale singers with smooth terms for the dependence on Survey Day, Hour, Moon Phase and Peak Power fitted for each of the MARUs; the plots show the estimated conditional dependence of humpback whale singer numbers on: Survey Day, the number of days since the first singing activity was recorded (x-axis), for (A) MARU 1 and (B) MARU 2; and Hour (x-axis), the diel cycle in hours, for (C) MARU 1 and (D) MARU 2. The y-axis, with scale is selected optimally for each plot, shows the contribution of the smooth term to the fitted values. Estimates (solid lines) and 95% confidence bands (dashed lines), with a rug plot indicating the covariate values of observations (short vertical bars along each x-axis), are shown. (TIF) [file pone.0086464.s003.tif]

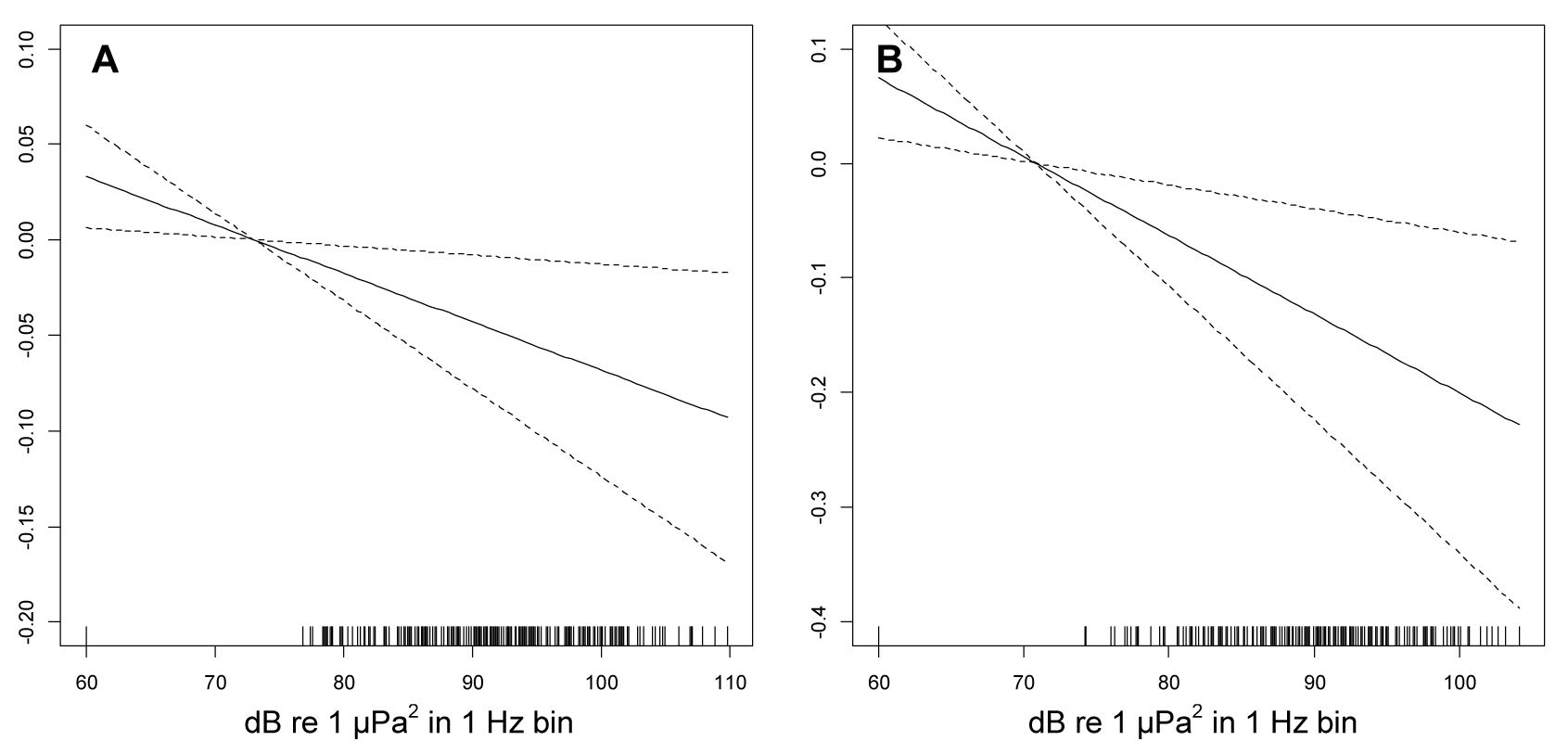

Supplement: Figure S4 — Impact of seismic survey pulse RL on humpback whale singing activity for the reduced dataset. Generalized Additive Mixed Models of the number of humpback whale singers with smooth terms for the dependence on Hour, Peak Power and Moon Phase for MARU 1 and only Peak Power for MARU 2, when restricting data to the first period of seismic activity 5–31 July 2008. Shown is the estimated conditional dependence of humpback whale singer numbers on Peak Power for (A) MARU 1 and (B) MARU 2. The x-axis in each plot shows Peak Power, describing received level of seismic survey pulse (in dB re: 1 µPa2 in a 1 Hz frequency bin) with a rug plot (short vertical bars) indicating the Peak Power values of observations. The y-axis, with scale selected optimally for each plot, shows the contribution of the smooth of Peak Power to the fitted values. Estimates (solid lines) are shown with 95% confidence bands (dashed lines). (TIF) [file pone.0086464.s004.tif]
